# ﻿*Penicillium* and *Talaromyces* diversity in cystic fibrosis patient sample and the description of a new species, *Penicillium subluteum* sp. nov. (Eurotiales, Aspergillaceae)

**DOI:** 10.3897/mycokeys.125.168897

**Published:** 2025-11-21

**Authors:** Ya Bin Zhou, Eelco F. J. Meijer, Tobias G. P. Engel, Qi Ming Wang, Zi Xuan Feng, Ferry Hagen, Martin Meijer, Bart Kraak, Jacques F. Meis, Jos Houbraken

**Affiliations:** 1 Westerdijk Fungal Biodiversity Institute, Utrecht, Netherlands Westerdijk Fungal Biodiversity Institute Utrecht Netherlands; 2 Department of Medical Microbiology and Immunology, Canisius-Wilhelmina Hospital(CWZ)/Dicoon, Nijmegen, Netherlands Canisius-Wilhelmina Hospital(CWZ)/Dicoon Nijmegen Netherlands; 3 Department of Medical Microbiology, Radboud University Medical Center, Nijmegen, Netherlands Radboud University Medical Center Nijmegen Netherlands; 4 Radboudumc-CWZ Center of Expertise for Mycology, Nijmegen, Netherlands Radboudumc-CWZ Center of Expertise for Mycology Nijmegen Netherlands; 5 School of Life Sciences, Institute of Life Sciences and Green Development, Hebei University, Baoding, Hebei, China Hebei University Baoding China; 6 Institute for Biodiversity and Ecosystem Dynamics, University of Amsterdam, Amsterdam, Netherlands University of Amsterdam Amsterdam Netherlands; 7 Department of Medical Microbiology, University Medical Center Utrecht, Utrecht, Netherlands University Medical Center Utrecht Utrecht Netherlands; 8 Institute of Translational Research, Cologne Excellence Cluster on Cellular Stress Responses in Aging-Associated Diseases (CECAD), Excellence Center for Medical Mycology (ECMM), University of Cologne, Cologne, Germany University of Cologne Cologne Germany

**Keywords:** Cystic fibrosis, diversity, new species, *
Penicillium
*, *Penicillium subluteum*, phylogeny, *Talaromyces*, taxonomy

## Abstract

*Penicillium* and *Talaromyces* species are frequently isolated from the respiratory tracts of cystic fibrosis (CF) patients, yet their diversity, ecological roles, and clinical significance remain poorly understood. In this study, we analyzed 521 fungal isolates (482 *Penicillium* and 39 *Talaromyces*) obtained from Dutch CF patients to investigate species diversity and prevalence. Using a combination of AFLP fingerprinting and DNA sequences analysis, we identified 57 *Penicillium* and 18 *Talaromyces* species, including a putatively new species named *Penicillium subluteum***sp. nov.** The most commonly isolated *Penicillium* species included *P. crustosum*, *P. frequentans*, *P. chrysogenum*, *P. rubens*, and *P. brevicompactum*, while *Talaromyces rugulosus* was the most prevalent *Talaromyces* species. Our findings highlight the underestimated diversity of *Penicillium* and *Talaromyces* in CF patients and emphasize the importance of accurate species identification for understanding fungal colonization patterns and assessing pathogenic potential. This study provides the most comprehensive overview to date of *Penicillium* and *Talaromyces* diversity in the CF airway and contributes valuable taxonomic and ecological insights into the role of these fungi in patients with chronic airway disease.

## ﻿Introduction

Cystic fibrosis (CF) is a genetic disorder caused by mutations in cystic fibrosis transmembrane conductance regulator (*CFTR*) gene, leading to impaired chloride ion transport across epithelial cell membranes. This results in thick, sticky mucus accumulation in the lungs, digestive tract, and other organs, predisposing patients to chronic respiratory infections and inflammation (Terlizzi and Lopes-Pacheco 2025). While bacterial pathogens like *Pseudomonas aeruginosa* and *Staphylococcus aureus* are commonly associated with CF lung infections, fungal organisms have emerged as significant contributors to the disease’s complexity and progression (Chesshyre et al. 2025).

Among the fungal taxa detected in CF respiratory samples, *Aspergillus fumigatus* has been most extensively studied due to its association with allergic bronchopulmonary aspergillosis and chronic colonization (Agarwal et al. 2013). However, non-*Aspergillus* fungi, particularly *Penicillium* and *Talaromyces* species, are increasingly identified in CF patients using culture-independent and molecular techniques (Pihet et al. 2009; Sudfeld et al. 2010; Chowdhary et al. 2014a; de Almeida et al. 2020; Hong et al. 2023). Although their clinical significance remains less well defined, these fungi are part of the broader mycobiome that may interact with the host immune system and co-infecting microbes to influence disease progression (de Almeida et al. 2020).

*Penicillium* species have historically been considered environmental contaminants or benign colonizers, but growing evidence suggests their potential role in airway inflammation (Rick et al. 2016). They are also commonly found in indoor and outdoor hospital air, as common as *Aspergillus* species (Asadzadeh et al. 2025). Some *Penicillium* species produce secondary metabolites, including mycotoxins and immunomodulatory compounds, which may exacerbate airway pathology or influence co-infections (Zhang et al. 2022). *Talaromyces* species, phylogenetically and phenotypically related to *Penicillium*, are also recognized more often in patients with CF. While some species such as *Talaromyces marneffei* are known pathogens in immunocompromised individuals (Pruksaphon et al. 2022), other species such as *T. amestolkiae* have been isolated from CF sputum and bronchoalveolar lavage samples (de Almeida et al. 2020). Their ecological roles and potential pathogenicity in CF remain underexplored, but they may be underestimated contributors to airway colonization or even infection, especially in patients receiving immunosuppressive therapy.

Given the frequent use of broad-spectrum antibiotics, corticosteroids, and other immunomodulatory treatments in CF care, fungal colonization—especially by less-characterized genera like *Penicillium* and *Talaromyces*—may be promoted and even selected. The ability of some of these fungi to form biofilms and resist azole antifungals further complicates their management (Siqueira and Lima 2012; Chowdhary et al. 2014b; Gauthier et al. 2023). As the CF mycobiome continues to be studied with high-throughput sequencing and improved culturing techniques, the importance of diverse fungal genera in CF lung disease is becoming increasingly apparent. In our previous study, we isolated numerous *Penicillium* and *Talaromyces* isolates from the respiratory samples of CF patients (Engel et al. 2019). However, many of these isolates were not identified to species level at that time. In the present study, we aim to identify these isolates more precisely and gain a better understanding of the relationship between *Penicillium*/*Talaromyces* and CF.

## ﻿Materials and methods

### ﻿Isolates

The isolates used in this study were collected between March 2010 and March 2013 from respiratory samples of CF patients attending five Dutch CF centers during routine quarterly visits, representing 82% of all Dutch CF patients. (Engel et al. 2019).

### ﻿DNA extraction, and identification

DNA extraction was performed as previously described (Engel et al. 2019). All identifications were carried out using Amplified Fragment Length Polymorphism (AFLP) fingerprinting, following established protocols (Kathuria et al. 2015). Each AFLP run included a set of reference isolates representing commonly encountered fungal pathogens to aid in species identification. When AFLP failed to yield a definitive identification, a partial *BenA* sequence was generated and used for sequence-based species identification (Visagie et al. 2014). Sequence similarity was assessed using the BLAST search program (NCBI, USA) to compare against sequences in GenBank. Only sequences from GenBank, the in-house database of the Westerdijk Fungal Biodiversity Institute, or those published in peer-reviewed journals were accepted to ensure accuracy. For isolates suspected to represent novel species, three additional loci (ITS, *CaM*, and *RPB2*) were sequenced for further phylogenetic analysis, following previously described methods (Zhou et al. 2024).

### ﻿Phylogenetic analysis

A phylogram was constructed based on a concatenated dataset of *BenA*, *CaM*, and *RPB2* gene sequences of *Penicillium* series *Paxillorum* species and related series. The ITS region was excluded, as it generally fails to resolve closely related *Penicillium* species and often results in poorly supported phylogenies (Visagie et al. 2014). An overview of the species included, along with their corresponding NCBI GenBank accession numbers, is provided in Suppl. material 1. Individual gene alignments were performed using MAFFT v. 7.427 (Katoh and Standley 2013), and the resulting alignments were combined into a three-locus dataset using SequenceMatrix (Vaidya et al. 2011). Phylogenetic analyses were conducted using both Maximum Likelihood (ML) and Bayesian Inference (BI) approaches. ML analysis was performed with RAxML-HPC2 on XSEDE v. 8.2.12 via the CIPRES Science Gateway (http://www.phylo.org) using the default GTRCAT model (Stamatakis 2014). MrModelTest v. 2.2 was used to determine the optimal nucleotide substitution model for each locus prior to Bayesian analysis (Nylander 2004). Bayesian inference was then performed using MrBayes v. 3.2.6, employing a Markov Chain Monte Carlo (MCMC) algorithm (Ronquist et al. 2012). Four simultaneous Markov chains were run for 10 million generations, sampling every 1,000 generations. The analysis was automatically halted when the average standard deviation of split frequencies fell below 0.01. The first 25% of the sampled trees were discarded as burn-in, and the remaining trees were used to calculate posterior probabilities (pp). The resulting phylogenetic trees were visualized with FigTree v. 1.4.2 and further annotated using Adobe Illustrator CS5. *P. sumatraense* CBS 281.36^T^ served as the outgroup.

### ﻿Morphological analysis

Macroscopic characteristics were assessed on various agar media, including Czapek yeast autolysate agar (CYA), CYA supplemented with 5% NaCl (CYAS), malt extract agar (MEA), yeast extract sucrose agar (YES), oatmeal agar (OA), creatine sucrose agar (CREA), and dichloran 18% glycerol agar (DG18) (Visagie et al. 2014). Isolates were inoculated at three points on 90 mm Petri dishes and incubated for 7 days at 25 °C in darkness. Additionally, CYA plates were incubated at 30 °C, 37 °C, and 40 °C for 7 days. After incubation, colony diameters were measured, and colony characteristics—including texture, degree of sporulation, obverse and reverse colors, production of soluble pigments, exudates, and ascomata—were assessed. Color descriptions followed the nomenclature of Kornerup and Wanscher (Kornerup and Wanscher 1967). Microscopic preparations were made from 1-week-old colonies grown on MEA. Lactic acid (60%) was used as the mounting medium, and 96% ethanol was applied to remove excess conidia and reduce air bubbles. Digital images were captured using a Zeiss Stereo Discovery V20 dissecting microscope (Carl Zeiss AG, Oberkochen, Germany) and a Zeiss AX10 Imager A2 light microscope (Carl Zeiss AG, Oberkochen, Germany), both equipped with Nikon DS-Ri2 cameras (Nikon Corporation, Tokyo, Japan) and NIS–Elements D v4.50 software (Nikon Corporation, Tokyo, Japan).

## ﻿Results

### ﻿Isolates

**Figure 1.**
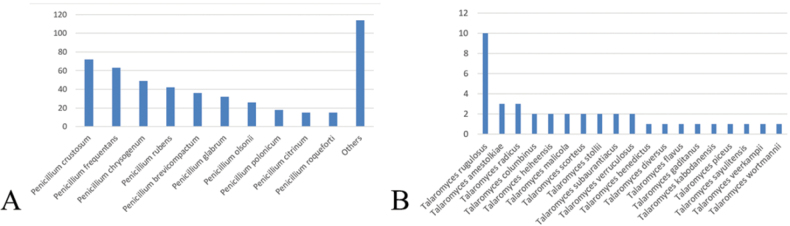
**A.** Diversity of *Penicillium* cultured in respiratory samples of CF patients; **B.** Diversity of *Talaromyces* cultured in respiratory samples of CF patients (X-axis shows the fungal species, Y-axis shows the number of isolates).

A total of 482 *Penicillium* and 39 *Talaromyces* isolates from 481 patients were included in this study (Suppl. material 2). Among these, we identified 57 *Penicillium* and 19 *Talaromyces* species. One *Penicillium* species positioned in *Penicillium* series *Paxillorum* are considered undescribed and potentially represent new taxa. A total of 303 strains were identified using AFLP analysis, including 38 *P. crustosum*, 31 *P. chrysogenum*, 30 *P. glabrum*, 30 *P. rubens*, 23 *P. brevicompactum*, and 21 *P. frequentans*, among others. The remaining strains were identified through *BenA* gene sequencing. All *BenA* sequences generated in this study have been deposited in GenBank under accession numbers PV646823–PV647039. Detailed identification information is provided in Suppl. material 2. The most frequently isolated *Penicillium* species were *P. crustosum* (n=72), *P. frequentans* (n=63), *P. chrysogenum* (n=49), *P. rubens* (n=42), *P. brevicompactum* (n=36), *P. glabrum* (n=32), *P. olsonii* (n=26), *P. polonicum* (n=18), *P. citrinum* (n=15), and *P. roqueforti* (n=15) (Fig. 1A). The most commonly isolated *Talaromyces* species was *T. rugulosus* (10 isolates), while all other *Talaromyces* species were represented by only 1 to 3 isolates each (Fig. 1B). Seventeen patients had repeat isolation of the same fungal species, including *P. frequentans* (n = 8), *P. crustosum* (n = 4), *P. chrysogenum* (n = 2), *P. olsonii* (n = 1), *P. albocoremium* (n = 1), and *T. rugulosus* (n = 1). Notably, six patients had three or more positive cultures with the same species, including *P. frequentans* (n = 3), *P. crustosum* (n = 2), and *P. albocoremium* (n = 1).

### ﻿Phylogeny

Although the ITS region is the primary fungal barcode, it generally fails to distinguish closely related species within *Penicillium*, and phylogenetic analyses based on ITS often yield poorly supported trees. Therefore, we conducted phylogenetic analyses using three single-locus datasets (*BenA*, *CaM*, and *RPB2*) as well as a concatenated dataset comprising these loci. In total, 68 strains from *Penicillium* series *Paxillorum* species and close series were included in the analysis. The combined alignment consisted of 2,079 characters: 453 bp for *BenA*, 577 bp for *CaM*, and 1,049 bp for *RPB2*. The best RAxML phylogram, with a final likelihood value of -8827.746066, is presented. The estimated base frequencies were: A = 0.241677, C = 0.252358, G = 0.248101, and T = 0.257864.

**Figure 2.**
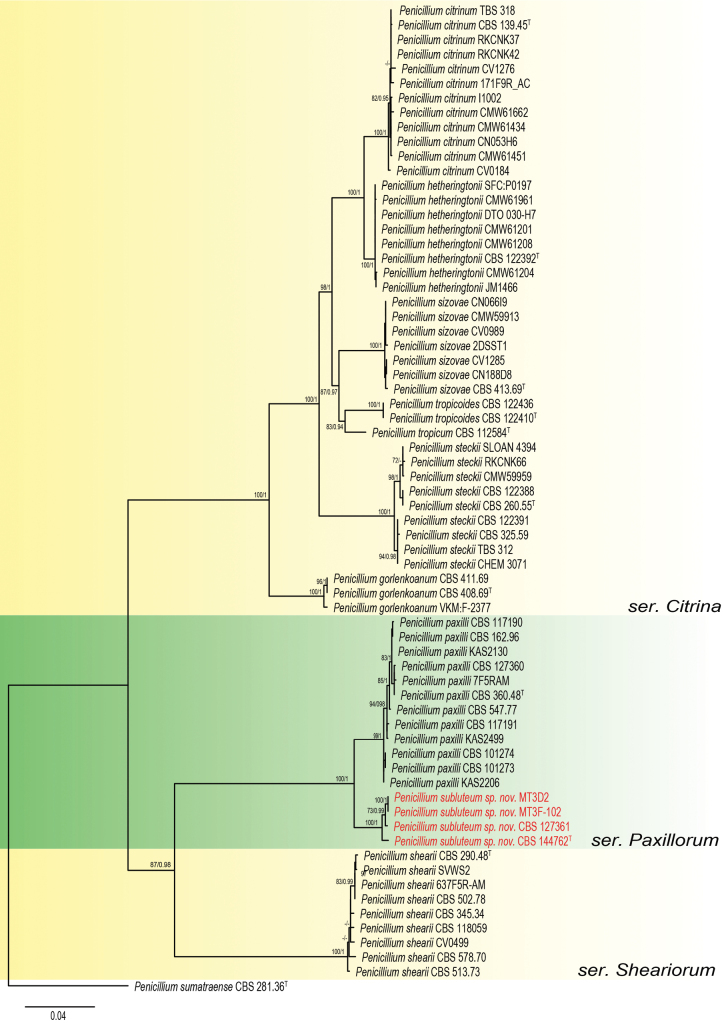
Phylogenetic trees based on a combined data set of *BenA*, *CaM* and *RPB2* sequences showing the relationship between *Penicillium* series *Paxillorum* species and related series. The BI posterior probability (pp) values and bootstrap percentages of the maximum likelihood (ML) analysis are presented at the nodes. Values less than 70% bootstrap support (ML) or less than 0.95 posterior probability (Bayesian analysis) are indicated with a hyphen or not shown. The bar indicates the number of substitutions per site. The phylogram is rooted with *P. sumatraense* CBS 281.36^T^.

Bayesian analyses were performed using the HKY+G substitution model for the *BenA* locus and the SYM+I+G model for both *CaM* and *RPB2*. The resulting phylogenetic tree (Fig. 2) shows that strains for the putative new species named *Penicillium subluteum* form a distinct and fully supported lineage (ML, 100%; BI, 1.00 posterior probability) sister to *P. paxilli*. Single-locus phylogenies based on the *BenA*, *CaM*, and *RPB2* datasets are presented in Suppl. material 3–5. In each of phylograms, *P. subluteum* is consistently positioned as a sister species to the *P. paxilli* clade, with strong statistical support (ML, 100%; BI, 1.00 posterior probability).

### ﻿Taxonomy

#### ﻿ Penicillium subluteum

Taxon classificationFungiEurotialesAspergillaceae

, Y.B. Zhou & Houbraken sp. nov.

3D100DCB-60C6-54B1-AD60-D102754B86EB

MB859221

Fig. 3

##### Etymology.

Name refers to its pale yellow reverse on CYA and DG18.

**Figure 3.**
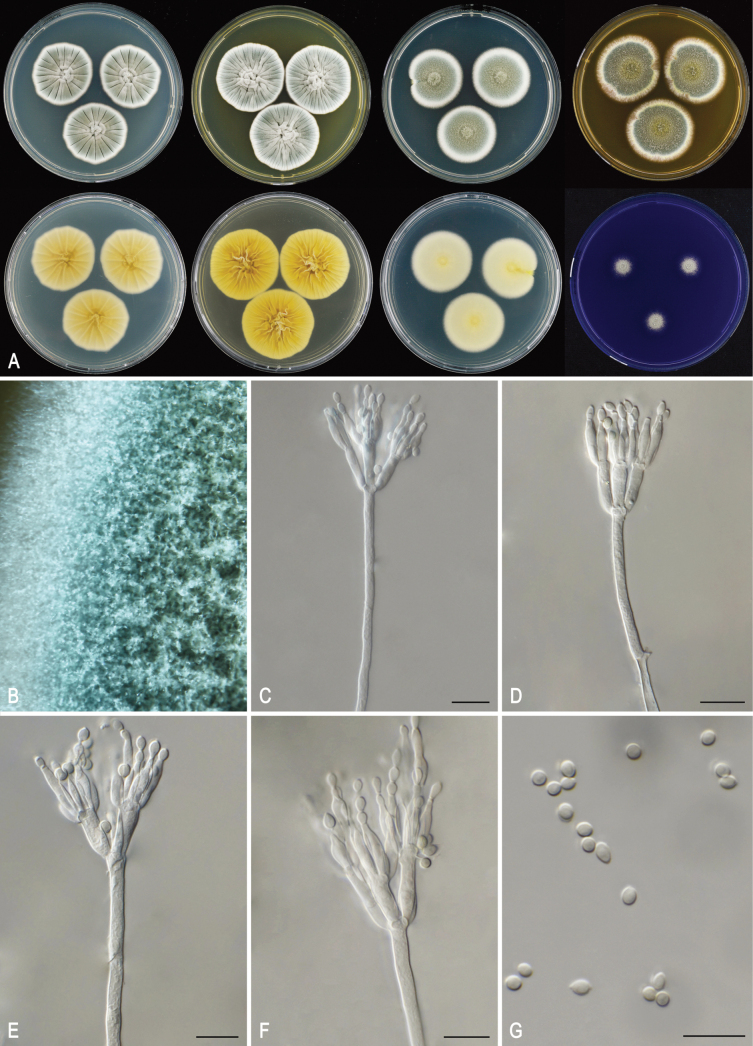
*Penicillium subluteum* CBS 144762^T^. **A.** Colony morphology of *P. subluteum* CBS 144762 incubated at 25 °C for 7 d: top row left to right, obverse CYA, obverse YES, obverse DG18 and obverse MEA; bottom row left to right, reverse CYA, reverse YES, reverse DG18 and CREA; **B.** Detail of colony on MEA after 7 d incubation; **C–F.** Conidiophores; **G.** Conidia. Scale bars: 10 μm.

##### Diagnosis.

Colonies grow rapidly on all tested media except CREA and exhibit a pale yellow reverse on CYA (3A3) and DG18 (1A3). Conidiophores are biverticillate and conidia are produced in long, distorted chains and are smooth and broadly ellipsoidal.

##### Classification.

Penicillium subgen. Aspergilloides sect. Citrina ser. Paxillorum

##### Typus.

**The Netherlands** • Nijmegen, human sputum; holotype: CBS H-23699; culture ex–type: CBS 144762 = DTO 200-G7 = CF0895.

##### ITS barcode.

PV645068 (alternative markers: *BenA* = PV646810; *CaM* = PV646812; *RPB2* = PV646822).

##### Colony diam.

(25 °C, 7 d, in mm): CREA 12–17; CYA 30–34; CYA 30 °C 17–20; CYA 37 °C no growth; CYA 40 °C no growth; CYAS 30–35; DG18 29–32; MEA 28–31; OA 20–28; YES 35–38.

##### Colony characters.

(25 °C, 7 d): CYA: Colonies slightly sunken in center, radial sulcate, margin nearly entire; sporulation dense; mycelium white; colony texture velvety; exudates present as hyaline droplets; soluble pigments absent; conidia *en masse* grayish green (26D5) to dull green (26E4); reverse pale yellow (3A3). *CYAS*: Colonies slightly sunken in center, radial sulcate, margin entire; sporulation dense; mycelium white; colony texture velvety; exudates present as hyaline droplets; soluble pigments absent; conidia *en masse* grayish green (26D5); reverse pale yellow (3A3). *MEA*: Colonies low, radial sulcate, margin entire; sporulation dense; mycelium white; colony texture velvety; exudates present as hyaline droplets; soluble pigments absent; conidia *en masse* grayish green (27E5); reverse grayish yellow (4B6). *YES*: Colonies sunken in center, radial sulcate, margin nearly entire; sporulation dense; mycelium white; colony texture velvety; exudates absent; soluble pigments absent conidia *en masse* grayish green (26D5) to dull green (26E4); reverse pale yellow (3A3). *DG18*: Colonies slightly raised in center, radial sulcate, margin entire; sporulation dense; mycelium white; colony texture velvety; exudates absent; soluble pigments absent; conidia *en masse* grayish green (26D6); exudates absent; soluble pigments absent; reverse pale yellow (1A3). *OA*: Colonies plane, margin entire; mycelium white; colony texture velvety; sporulation dense; conidia *en masse* grayish green (27D5). exudates present as hyaline droplets; soluble pigments absent.*CREA*: poor growth, acid production absent, base formation absent.

##### Micromorphology.

Conidiophores biverticillate; stipes smooth-walled or finely roughened, 130–325 × 2–4 µm; metulae cylindrical, 9.5–13 × 2.5–4.5 µm; phialides flask shaped, 3–8 per metulae, 8.5–12.5 × 2.5–3.5 µm; conidia in long, distorted chains, smooth, broadly ellipsoidal, 3–4 × 2.5–3.5 µm.

##### Additional specimens examined.

**Australia** • Queensland, Barrine, near lake Cratez, isolated from soil, CBS 127361 = DTO 030–A6 = IBT 29070; **China** • Tibet, Zunyi Area, Meti Country, isolated from soil, MT3F-102.

##### Note.

This species is both morphologically and phylogenetically related to *P. paxilli*. However, *P. subluteum* has a pale yellow reverse on CYA and DG18, in contrast to the pale reverse observed in *P. paxilli* (Houbraken et al. 2011). Pairwise nucleotide differences between the type strains of the two species include 10 nucleotides in ITS, 19 bp in *BenA*, 26 bp in *CaM*, and 30 bp in *RPB2*.

## ﻿Discussion

This study revealed a surprisingly diverse assemblage of *Penicillium* and *Talaromyces* species isolated from respiratory samples of Dutch cystic fibrosis (CF) patients. The predominance of specific *Penicillium* and *Talaromyces* species among isolates from Dutch CF patients, especially *P. crustosum*, *P. frequentans*, *P. chrysogenum*, *P. rubens*, *P. brevicompactum*, *P. glabrum*, *P. olsonii*, *P. polonicum*, *P. citrinum*, *P. roqueforti*, and *T. rugulosus*is likely multifactorial. Several interrelated factors likely contribute to their frequent isolation.

The frequent detection of these species in respiratory samples may reflect their environmental distribution. The predominant species *P. chrysogenum* and *P. rubens* are common in water-damaged buildings because these species grow on both wet and semi-dry materials (Andersen et al. 2011; van Laarhoven et al. 2015; Reboux et al. 2019). Notably, these species also demonstrate moderate growth at 37 °C (Guevara-Suarez et al. 2016), potentially enabling survival and growth in human airways where local microenvironments may be cooler than core body temperature. Their ability to tolerate osmotic stress and nutrient limitation may further enhance persistence in the CF lung’s unique ecological niche (Frisvad and Samson 2004). The frequent use of antimicrobial therapy/ prophylaxis and immunosuppressants in CF management likely creates selective pressures favoring fungal colonization (Beswick et al. 2020). While most *Penicillium* and *Talaromyces* species remain susceptible to antifungals, some exhibit reduced azole sensitivity (Chowdhary et al. 2014b; Singh et al. 2021), potentially allowing persistence during *Aspergillus*-targeted therapy.

An important consideration in our study is whether all cultured fungi represent true colonization of the respiratory tract. The majority of *Penicillium* and *Talaromyces* species were isolated only once per patient. This raises the possibility that many positive cultures reflect the presence of inhaled fungal spores rather than active, sustained colonization. Inhaled spores, when trapped in mucus, can be cultured in the laboratory without actual hyphal growth in the airways. This interpretation is supported by our previous findings, which demonstrated marked year-to-year variation in the detection of specific fungal species in CF respiratory samples (Engel et al. 2019). Some fungi were isolated multiple times in one year but rarely or not at all in other years, suggesting transient environmental exposure rather than persistent colonization (Engel et al. 2019).

However, we also observed potential evidence of ongoing fungal presence in a subset of patients. Seventeen patients had repeat isolation of the same fungal species and six patients had three or more positive cultures with the same species, including one individual with *P. crustosum* isolated five times. These findings suggest that, at least in some cases, *Penicillium* and *Talaromyces* species may persist in the airways of CF patients and potentially establish colonization.

Although *Talaromyces* species are generally known for their higher thermotolerance compared to *Penicillium* (Guevara-Suarez et al. 2016), our study revealed a higher number of *Penicillium* isolates in respiratory samples from CF patients. This discrepancy likely reflects the complex temperature microenvironments within the human airway, which often remain below core body temperature—especially in upper and central regions. Studies using direct thermistor probes show that during quiet breathing, airway temperatures range from approximately 32.0 °C in the upper trachea to 35.5 °C in the subsegmental bronchi (McFadden et al. 1985). These cooler microclimates may favor the growth or persistence of cooler-adapted fungi such as *Penicillium* spp. A notable example is *P. crustosum*, which is unable to grow at 37 °C under laboratory conditions (Guevara-Suarez et al. 2016), yet was repeatedly isolated from the same CF patient on five separate occasions. This suggests that *P. crustosum* may survive and potentially colonize airway niches where temperatures remain below the threshold required to inhibit its growth. Observed persistence likely reflects adaptation to airway microclimates rather than transient environmental exposure.

*P. crustosum* is a generalist, and can cause rot of pomaceous fruits, but is also associated with food (nuts, oilseeds, dried meat, corn, rice, cheese) (Raper and Thom 1949; Frisvad 1981), soil, cardboard, leather, textiles, wood and indoor air (Frisvad and Samson 2004; Samson et al. 2019). The high incidence of *P. olsonii* (n=26) is noteworthy. In indoor environments, this species thrives particularly well in potting soil (Frisvad and Samson 2004) or mouldy coffee grounds (J. Houbraken, personal observation). In addition to commonly occurring indoor species, several isolates showed strong associations with specific substrates. For example, multiple species typically associated with causing rot of bulbs and roots were detected: *P. albocoremium* (n=5, onions), *P. allii* (n=7, garlic), *P. tulipae* (n=1, tulip bulbs), *P. venetum* (n=1, Iris, Aspargus) (Dugan and Strausbaugh 2019). Other isolates were associated with fruit rot, such as *P. digitatum* (n=4) and *P. italicum* (n=1) from citrus fruit, and *P. expansum* (n=9) from apples (Papoutsis et al. 2019; Luciano-Rosario et al. 2020). The presence of *P. roqueforti* (n=15) may be explained by exposure to conidia from Roquefort or other blue-veined cheeses, but can also originate from other sources such as silage (Ropars et al. 2017). These findings highlight the importance of correct species identification—not only for treatment, but also for prevention. Accurate identification provides ecological insights and can help trace and eliminate potential sources that may impact patient health.

Our description of the novel species *P. subluteum* highlights ongoing taxonomic challenges in fungal identification. Although ITS sequences can differentiate *P. subluteum* from some closely related species, they are insufficient for resolving other species in this section such as *P. tropicum* and *P. tropicoides* in phylogenetic analyses (data not shown), due to relatively low nucleotide divergence. Therefore, we recommend sequencing of *BenA, CaM*, or *RPB2* genes for the accurate identification of *P. subluteum*. Historically, many *Penicillium* and *Talaromyces* isolates were likely misclassified or dismissed as contaminants due to limited diagnostic methods (Mortensen et al. 2011). Advanced molecular techniques now reveal greater diversity than previously recognized, as evidenced by our identification of 75 distinct species. *T. rugulosus*, in particular, may have been underreported due to phenotypic similarities with *Penicillium* species (Yilmaz et al. 2014).

Besides the commonly encountered species, and the introduction of *P. subluteum* here, we also identified several species described after the sampling period (2010–2013). These include *P. lentum*, *P. longicatenatum*, *P. rudallense*, *P. speluncae*, *T. benedictus*, *T. heiheensis*, *T. kabodanensis*, *T. malicola* and *T. subaurantiacus*. Although recently described, this does not imply these species are rare. For instance, database searches showed that *P. subluteum* has also been isolated from Australia and China. Similarly, *P. ausonanum* (CF2909, CF0089) appears to be relatively common and was previously isolated from various substrates in the Netherlands, such as compost, soil, and ladybugs (J. Houbraken, personal observations).

While the pathogenicity of these fungi remains uncertain, their frequent isolation in CF patients warrants attention in clinical management. Potential implications include contributions to chronic inflammation through mycotoxin production or immune modulation, interactions with bacterial pathogens that may influence disease progression, and the development of antifungal resistance under prolonged therapeutic pressure.

This research has several limitations. First, the single country design may limit generalizability to other CF populations. Second, the lack of longitudinal clinical data prevents assessment of species-specific disease impacts. Third, while we used advanced molecular methods, some isolates may represent novel taxa requiring further characterization. Additionally, we did not evaluate fungal load, viability, or antifungal susceptibility, nor examine potential fungal-bacterial interactions. These gaps highlight important areas for future research.

## ﻿Conclusion

In conclusion, our findings demonstrate that *Penicillium* and *Talaromyces* species are diverse and frequently present in CF airways, with certain species showing potential adaptations to this environment. While their clinical significance requires further investigation, these fungi should no longer be automatically dismissed as contaminants. Improved understanding of their ecological and pathogenic potential may lead to better management strategies for CF patients with fungal colonization. Our description of *P. subluteum* sp. nov. provides a crucial taxonomic reference for future identifications, advancing understanding of fungal biodiversity.

## Supplementary Material

XML Treatment for Penicillium subluteum

## ﻿Additional information

### Use of AI

No use of AI was reported.

### Author ORCIDs

Ya Bin Zhou https://orcid.org/0000-0001-5775-3861

Eelco F.J. Meijer https://orcid.org/0000-0002-0226-024X

Jacques F. Meis https://orcid.org/0000-0003-3253-6080

Jos Houbraken https://orcid.org/0000-0003-4893-4438

### Data availability

All of the data that support the findings of this study are available in the main text or Supplementary Information.

## Supplementary materials

Supplementary material 1 Strains used for the phylogentic analysis Data type xlsx https://binary.pensoft.net/file/1472424This dataset is made available under the Open Database License (http://opendatacommons.org/licenses/odbl/1.0/). The Open Database License (ODbL) is a license agreement intended to allow users to freely share, modify, and use this Dataset while maintaining this same freedom for others, provided that the original source and author(s) are credited.Ya Bin Zhou, Eelco F.J. Meijer, Tobias G.P. Engel, Qi Ming Wang, Zi Xuan Feng, Ferry Hagen, Martin Meijer, Bart Kraak, Jacques F. Meis, Jos Houbraken

Supplementary material 2 Isolates list in this study Data type xlsx https://binary.pensoft.net/file/1472425This dataset is made available under the Open Database License (http://opendatacommons.org/licenses/odbl/1.0/). The Open Database License (ODbL) is a license agreement intended to allow users to freely share, modify, and use this Dataset while maintaining this same freedom for others, provided that the original source and author(s) are credited.Ya Bin Zhou, Eelco F.J. Meijer, Tobias G.P. Engel, Qi Ming Wang, Zi Xuan Feng, Ferry Hagen, Martin Meijer, Bart Kraak, Jacques F. Meis, Jos Houbraken

Supplementary material 3 Phylogenetic trees based on a combined data set of *BenA* sequences showing the relationship between *Penicillium* series *Paxillorum* species and related series Data type jpg https://binary.pensoft.net/file/1472426This dataset is made available under the Open Database License (http://opendatacommons.org/licenses/odbl/1.0/). The Open Database License (ODbL) is a license agreement intended to allow users to freely share, modify, and use this Dataset while maintaining this same freedom for others, provided that the original source and author(s) are credited.Ya Bin Zhou, Eelco F.J. Meijer, Tobias G.P. Engel, Qi Ming Wang, Zi Xuan Feng, Ferry Hagen, Martin Meijer, Bart Kraak, Jacques F. Meis, Jos Houbraken

Supplementary material 4 Phylogenetic trees based on a combined data set of *CaM* sequences showing the relationship between *Penicillium* series *Paxillorum* species and related series Data type jpg https://binary.pensoft.net/file/1472427This dataset is made available under the Open Database License (http://opendatacommons.org/licenses/odbl/1.0/). The Open Database License (ODbL) is a license agreement intended to allow users to freely share, modify, and use this Dataset while maintaining this same freedom for others, provided that the original source and author(s) are credited.Ya Bin Zhou, Eelco F.J. Meijer, Tobias G.P. Engel, Qi Ming Wang, Zi Xuan Feng, Ferry Hagen, Martin Meijer, Bart Kraak, Jacques F. Meis, Jos Houbraken

Supplementary material 5 Phylogenetic trees based on a combined data set of *RPB2* sequences showing the relationship between *Penicillium* series *Paxillorum* species and related series Data type jpg https://binary.pensoft.net/file/1472428This dataset is made available under the Open Database License (http://opendatacommons.org/licenses/odbl/1.0/). The Open Database License (ODbL) is a license agreement intended to allow users to freely share, modify, and use this Dataset while maintaining this same freedom for others, provided that the original source and author(s) are credited.Ya Bin Zhou, Eelco F.J. Meijer, Tobias G.P. Engel, Qi Ming Wang, Zi Xuan Feng, Ferry Hagen, Martin Meijer, Bart Kraak, Jacques F. Meis, Jos Houbraken
