# Supplementary figures and images for: ﻿Penicillium and Talaromyces diversity in cystic fibrosis patient sample and the description of a new species, Penicillium subluteum sp. nov. (Eurotiales, Aspergillaceae)
Source: MycoKeys. 2025 Nov 21;125:263–78. doi: 10.3897/mycokeys.125.168897 (PMC12663725; doi:10.3897/mycokeys.125.168897)

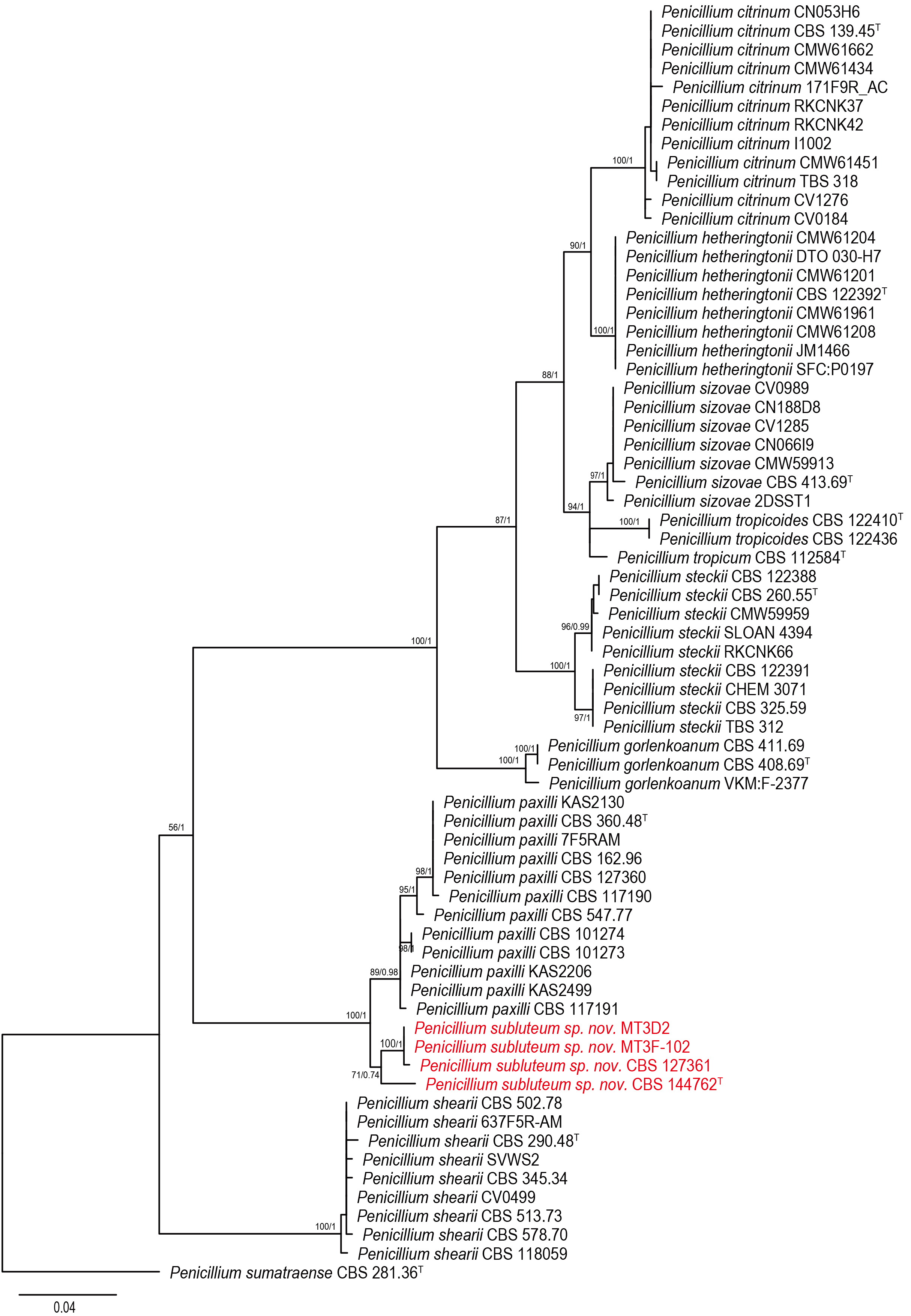

Supplement: Supplementary material 3 — Phylogenetic trees based on a combined data set of BenA sequences showing the relationship between Penicillium series Paxillorum species and related series [file mycokeys-125-263-s003.jpg]

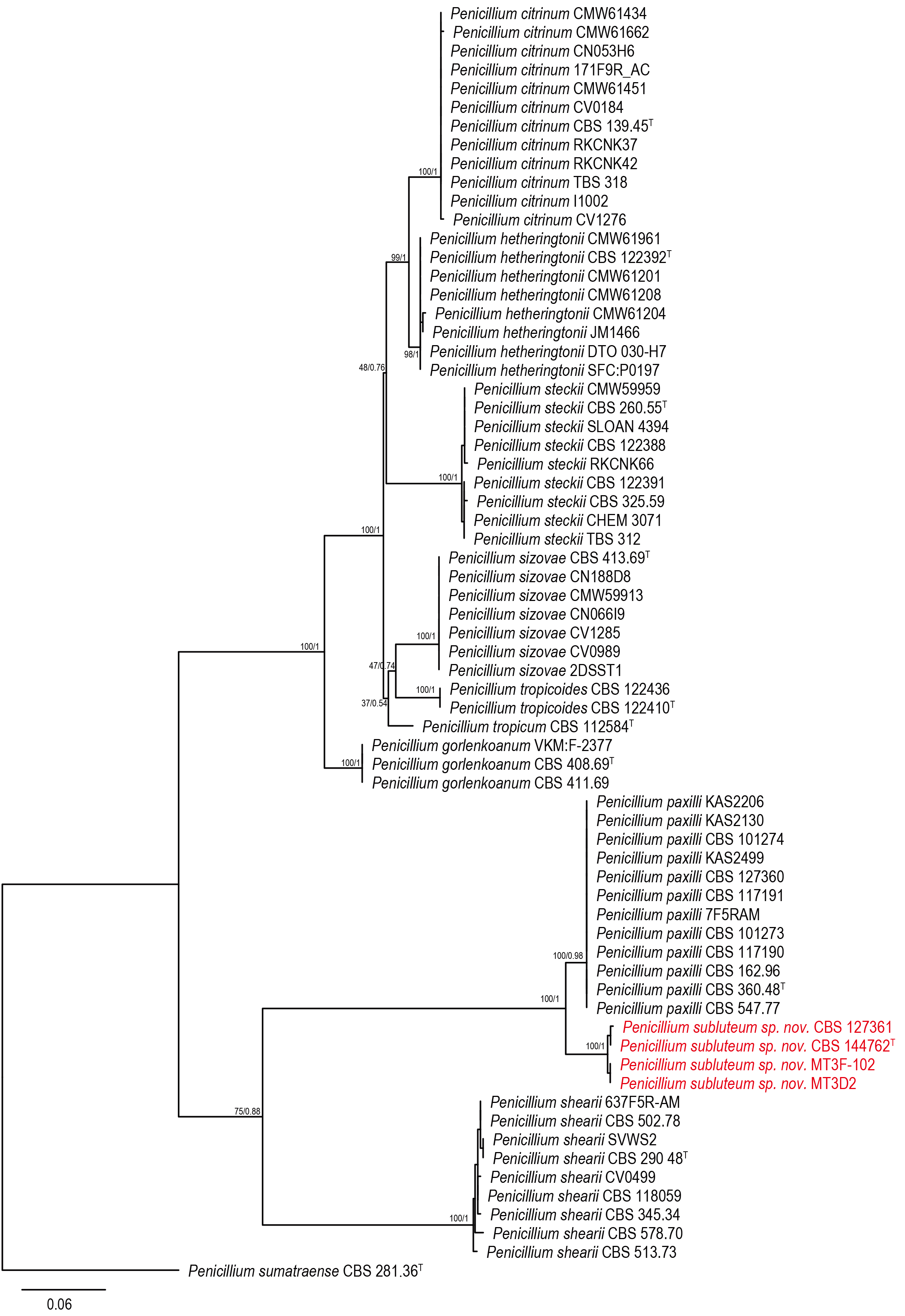

Supplement: Supplementary material 4 — Phylogenetic trees based on a combined data set of CaM sequences showing the relationship between Penicillium series Paxillorum species and related series [file mycokeys-125-263-s004.jpg]

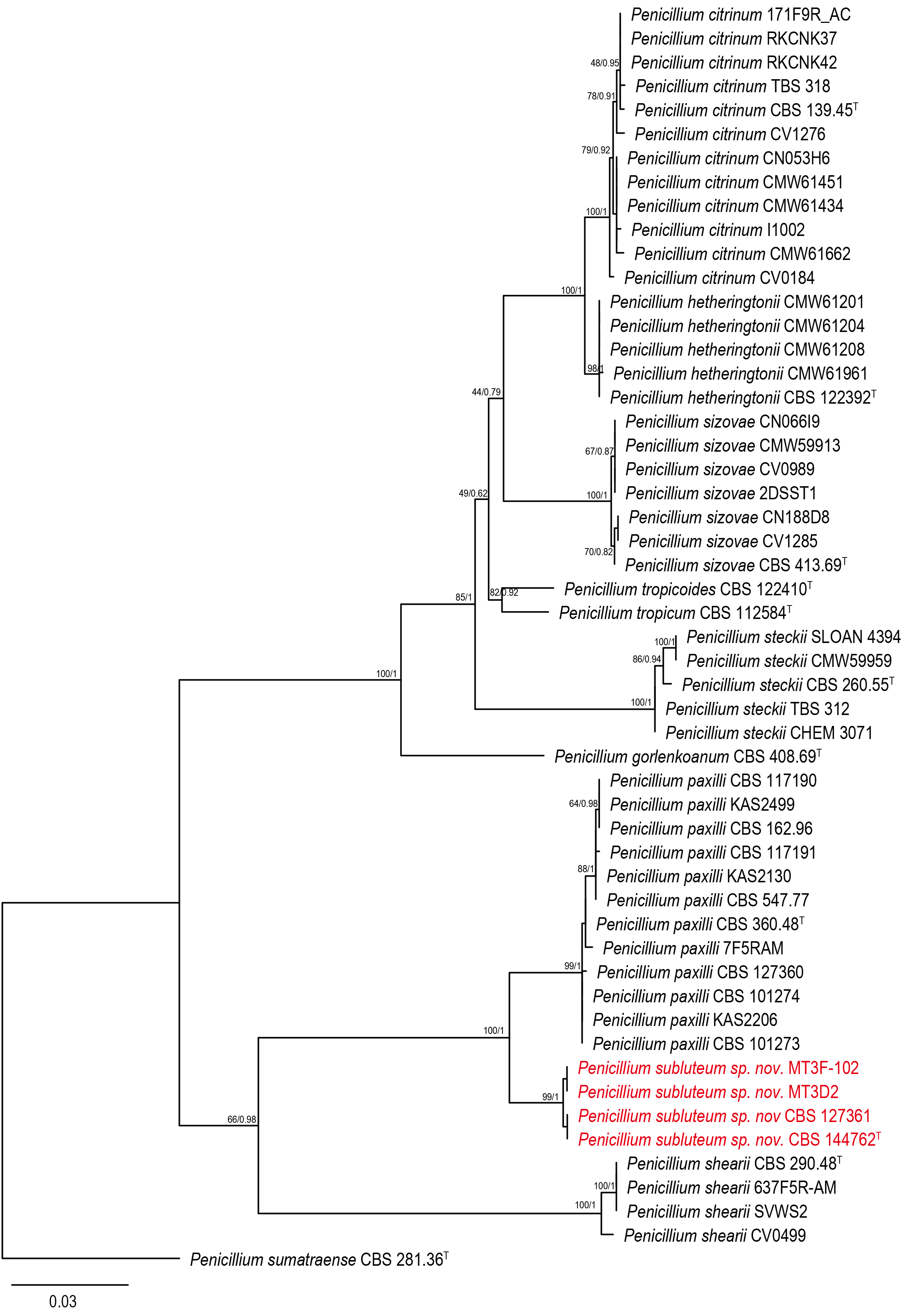

Supplement: Supplementary material 5 — Phylogenetic trees based on a combined data set of RPB2 sequences showing the relationship between Penicillium series Paxillorum species and related series [file mycokeys-125-263-s005.jpg]
